# The National Program of Cancer Registries: Explaining State Variations in Average Cost per Case Reported

**Published:** 2005-06-15

**Authors:** Hannah K Weir, Gregory D Berg, Edward C Mansley, Kimberly A Belloni

**Affiliations:** Division of Cancer Prevention and Control, National Center for Chronic Disease Prevention and Health Promotion (NCCDPHP), Centers for Disease Control and Prevention (CDC); McKesson Health Solutions, McKesson Corp, Broomfield, Colo; Outcomes Research & Management, Merck & Co, Inc, West Point, Pa; Division of Cancer Prevention and Control, NCCDPHP, CDC, Atlanta, Ga

## Abstract

**Introduction:**

The Centers for Disease Control and Prevention's National Program of Cancer Registries is a federally funded surveillance program that provides support and assistance to state and territorial health departments for the operation of cancer registries. The objective of this study was to identify factors associated with the Centers for Disease Control and Prevention's costs to report cancer cases during the first 5 years of the National Program of Cancer Registries.

**Methods:**

Information on expenditures and number of cases reported through the National Program of Cancer Registries was used to estimate the average cost per case reported for each state program. Additional information was obtained from other sources, and regression analyses were used to assess the contribution of each factor.

**Results:**

Average costs of the National Program of Cancer Registries differed substantially among programs and were inversely associated with the number of cases reported (*P* < .001). The geographic area of the state was positively associated with the cost (*P *= .01), as was the regional cost of living (*P* = .08), whereas the program type (i.e., enhancement or planning) was inversely associated with cost (*P* = .08).

**Conclusion:**

The apparent existence of economies of scale suggests that contiguous state programs might benefit from sharing infrastructure and other fixed costs, such as database management resources, depending on the geographic area and population size served. Sharing database management resources might also promote uniform data collection and quality control practices, reduce the information-sharing burden among states, and allow more resources to be used for other cancer prevention and control activities.

## Introduction

### Cancer incidence data

The National Institutes of Health (NIH) estimated that in 2003, the annual direct and indirect costs of cancer were approximately $189.5 billion (1). Despite the recent reports of a decrease in age-adjusted cancer death rates (2-5), costs are likely to increase. Because of the growth and the aging of the U.S. population, the total number of cancer cases is estimated to double by 2050 if current incidence rates remain stable (6). However, effective prevention and treatment measures exist that could substantially reduce the number of cases and prevent many cancer-related deaths (7). To reduce the cancer burden, particularly among medically underserved populations, high-quality screening and treatment services must be available and accessible to all segments of the U.S. population (8). Cancer incidence data from population-based cancer registries will become increasingly more important for identifying and targeting populations at high risk for developing cancer. In addition, these data are helpful for monitoring progress toward meeting cancer prevention and control goals such as those established by *Healthy People 2010* (9).

Data from the Centers for Disease Control and Prevention's (CDC's) federally funded state- or territory-managed surveillance programs, including the National Program of Cancer Registries (NPCR), are routinely used to report the incidence (i.e., new occurrence) of diseases and track the health of the U.S. population (10-12). The operating efficiency of these surveillance programs can be examined by comparing the various programs' average cost to report a case of disease. A comparison of average costs of these programs, in addition to an exploration of variations in average costs, can help guide current and future program planning and evaluation. 

The purpose of our analysis was to gain insight into factors associated with the average NPCR cost to report an incident case of cancer through population-based cancer registry programs funded during the NPCR's first 5 years.

### Background of the National Program of Cancer Registries

Before the NPCR was established, many states did not have a cancer registry, and the states that did generally lacked the resources and legislative support to collect high-quality data that were complete and timely (13). In 1992, the U.S. Congress recognized the need for more local and state cancer data and passed the Cancer Registries Amendment Act (Public Law 102-515) (14), which authorized the CDC to establish the NPCR. In 1994, the NPCR began providing financial support and technical assistance to state health departments for the operation of statewide, population-based cancer registries. State health departments or their authorized designees became eligible for one of two categories of funding. The *enhancement* category of funding supported the operation of existing cancer registries. Enhancement programs continued to receive their current level of state funding (i.e., the funding they received at the time of initial funding from the CDC) and contributed, or matched, $1 of state funds for every $3 of federal funds received. Matching funds were financial or direct (i.e., in-kind) assistance. The second category of funding involved planning and implementing a new cancer registry in a state with no previous registry. These *planning* programs were not required to match federal funds with state funds.

After the first program announcement (15) in 1994 and the approval of a congressional appropriation of $16.8 million, 42 states and the District of Columbia were awarded funds (34 enhancement programs and 9 planning programs). After the second program announcement (16) in 1997, three additional states and three territories were awarded funds (two enhancement programs and four planning programs). As of 1999, the last year of the NPCR's first 5-year period, with a congressional appropriation of $24.3 million, 45 states, the District of Columbia, and 3 territories were receiving federal support for the operation of population-based cancer registries.

Cancer registries in the remaining five states (Connecticut, Hawaii, Iowa, New Mexico, and Utah) receive federal support from the National Cancer Institute's (NCI's) Surveillance, Epidemiology, and End Results (SEER) Program (17). The SEER Program also operates six cancer registries in metropolitan areas (Atlanta, Detroit, Los Angeles, San Francisco–Oakland, Seattle–Puget Sound, and San Jose–Monterey) and collects data on special (i.e., minority) populations in Alaska, Arizona, and Georgia. SEER metropolitan-area and special-population cancer registries report incidence data to NCI and the NPCR-funded statewide cancer registries in their respective states.

NPCR-funded cancer registries are required to collect and report information on all state residents who are diagnosed or treated with in situ or invasive cancer, including residents who are diagnosed and treated outside their state of residence. In 2000, the CDC began to receive, evaluate, and publish data from participating cancer registry programs (18). NPCR registries are required to report their incidence data annually beginning with the reference year, which is the first year in which data was collected with the assistance of NPCR funds. The reference year varies among NPCR programs according to the year the state began receiving federal funds and the amount of time required to plan and implement a program to begin collecting and reporting cancer incidence data.

To promote standardization among state programs, cancer registries collect and report data using uniform codes and procedures established by the North American Association of Central Cancer Registries (NAACCR) (19). The SEER Program and the NPCR work closely with the NAACCR to promote the collection and publication of high-quality cancer incidence data; high-quality data meet national data standards for completeness of case ascertainment, timeliness, and quality (20,21). In 2002, in collaboration with the NAACCR, the CDC and the NCI began publishing the *United States Cancer Statistics* surveillance reports. The reports comprise data that have been reported to the two federally funded programs (SEER and NPCR) and have met evaluation criteria developed by the NAACCR to identify registries with high-quality data (10,11).

## Methods

### Eligible programs

**Figure 1:**
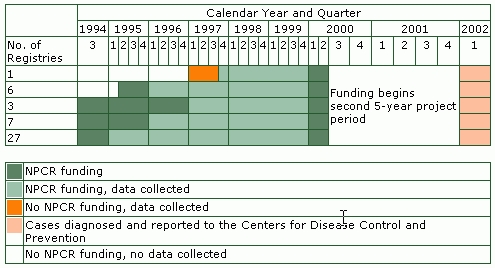
Federal funding and data collection and reporting activities of state cancer registries supported by the National Program of Cancer Registries (NPCR) during the first 5-year project period. Data from 43 states and the District of Columbia are included.

Figure 1 provides information about data collection and reporting by NPCR-funded state cancer registries during the project's first 5-year period. Thirty-seven programs began receiving funds in 1994 — 27 to report on cases beginning in 1995, 7 to report on cases beginning in 1996, and 3 to report on cases beginning in 1997. Six additional programs received funds in May 1995 to report on cases beginning in 1996. One additional program received funds in 1997 so that it could begin reporting cases in 1997.

To be eligible for inclusion in this analysis, state health departments or their designees 1) had to have received federal funds from the NPCR and 2) by 2002, had to have collected and reported cancer incidence data to the CDC for at least 2 consecutive years. The analysis was restricted to 43 state programs and the District of Columbia (referred to collectively as *states*). Information on territory-managed programs was not available for this analysis. The remaining two states (South Dakota and Tennessee) and three territories (Puerto Rico, the Republic of Palau, and the Virgin Islands) received funding beginning in 1997 and 1998 and began reporting data beginning in 1999 or 2000, so they were not eligible for inclusion in this analysis.

### Cases registered

Each January, the cancer registries report their cumulative incidence data to the CDC, beginning with their reference year. For this analysis, we used the total number of incident cases (in situ and invasive) diagnosed from the state's reference year through 1999 (as reported to the CDC in January 2002, approximately 24 months after the close of the 1999 diagnosis year) (Figure 1). The diagnosis year is the calendar year in which the cancer case was diagnosed.

### National Program of Cancer Registries costs

We obtained the amounts of federal funds allocated to state health departments (or their designees) from the Grant Management Information System (GMIS) maintained by the CDC. GMIS contains information on federal funds that were available to each state program at the beginning of each grant period and the amount that remained unused at the end. We calculated the NPCR cost for each year by subtracting the amount of unused funds from the amount awarded. We converted these costs to year 2000 dollars using the U.S. consumer price index (CPI) (22). Grant periods were from October through September of the following year, with the exception of six state programs that received their initial funding beginning in May 1996. In addition, in the last year of funding (1999), the states received a 9-month cost extension that ended on June 30, 2000 (Figure 1).

### National Program of Cancer Registries cost per case reported

Registries are expected to report high-quality incidence data to the CDC approximately 24 months after the close of each diagnosis year. However, resources spent in a given grant period cannot be apportioned to the year cases were diagnosed because in any given grant period, registries can use a portion of their federal funds to collect current incidence data and use the remaining funds to prepare previous years' incidence data for reporting to the CDC. For example, federal funds from grant periods in the October 1998 to June 2000 time frame were used to collect and report data for cases diagnosed in 1999.

Because we could not determine how much federal money from each grant period was used to report incident cases for a particular diagnosis year, we decided to calculate the average monthly funds spent in the period that 1) began with each program's reference month and year and 2) ended on June 30, 2000. We multiplied this amount by 12 to determine the average annual federal funds spent. We estimated the average number of cases diagnosed each year by adding the total number of cases reported, beginning with the program's reference year and ending with 1999 incident cases. We determined the average annual number of reported cases by dividing the total number of cases by the number of diagnosis years as reported to the CDC. Dividing the average annual federal funds spent by the average annual number of cases reported yielded an estimate of the average annual NPCR cost to report an incident case of cancer for each NPCR-funded  state surveillance program. Before collecting and reporting data, planning programs had a 1- to 2-year start-up period in which they planned and implemented their cancer registries. Because no output measures (i.e., number of cases) were tracked during the start-up period, we did not consider the amount of federal funds spent during this time when calculating average NPCR costs for planning programs.

### Explanatory factors

Scatter plots suggested a curvilinear relationship between average cost (the dependent variable) and number of cases reported (the output levels), so we used log–log transformations of these variables. In addition to output levels, we examined other registry characteristics, including the way in which the funding was administered (i.e., by the state health department or a designee); whether the state program was an existing (enhancement) or a new (planning) program; whether the state program had high-quality data, such as the 1999 incidence data that were published in the *United States Cancer Statistics: 1999–2001 Incidence and Mortality* report (10); and whether the state had a SEER metropolitan-area cancer registry (Georgia, Michigan, California, and Washington), a special-population cancer registry (Arizona, Alaska, and Georgia), or both (Georgia) operating within its catchment area. We also considered the following state characteristics in the analysis: the cost of living as estimated by the 2000 regional CPI (22), the state's composition (urban or rural, expressed as the percentage of the population that resided in an urban area) (23), and the geographic area of the state (expressed per 1000 sq miles) (24). To estimate the cost of living, we assigned the 2000 regional CPIs (for the West, Midwest, South, and Northeast) to the state program (22) and treated the values as continuous variables for the purpose of analysis.

### Statistical analysis

We used linear regression analyses to assess the relationship between the log average NPCR cost per case reported and explanatory factors. Using SAS, version 8 (SAS Institute Inc, Cary, NC), we performed multivariate modeling with backward elimination (25). We fit a full model (with all variables included) and assessed the contribution of each individual variable separately while simultaneously adjusting for all other specified variables. We sequentially eliminated variables until the final (parsimonious) model contained only variables with a *P* value of .10 or less. Because of the relatively small number of observation units (n = 44) and the potential for confounding, we used a less conservative *P* value so that we would not eliminate potentially important explanatory factors. We assessed the goodness of fit of the model by examining the adjusted R^2^ value. Subset analyses, in which the best models contained one, two, three, or more variables, were used to verify the model selection results (26). We used the parsimonious model to predict the average NPCR cost per case reported for each NPCR registry.

## Results

**Figure 2:**
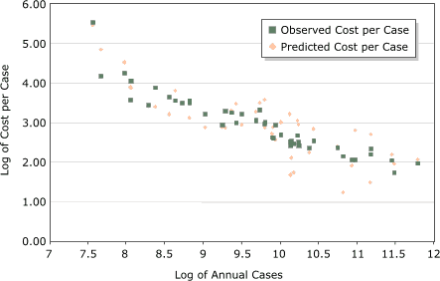
Observed and predicted log average National Program of Cancer Registries (NPCR) cost per case reported compared with the log of number of cases reported for programs participating in the first 5-year funding period of the program. Model contained average annual number of cancer cases reported, area (per 1000 sq miles), NPCR program type (enhancement or planning), and the 2000 consumer price index.

The average annual federal funds spent, the average annual number of cases reported, and the average NPCR cost per case reported varied substantially among state programs (Table 1; Figure 2). The mean average annual federal funds spent by an NPCR-funded program was $382,698, and the median was $312,698, with a range of $120,464 (Delaware) to $1,080,370 (Texas). The mean average number of annual cases (in situ and invasive) reported by an NPCR-funded program was 27,353, and the median was 18,999, with a range of 1929 (Alaska) to 132,525 (California) cases per year. The mean average annual NPCR cost per case reported for all programs was $29.20, and the median was $18.43, with a range of $3.45 (Michigan) to $234.52 (Alaska).

Table 2 shows the distribution of explanatory variables among cancer registries. Nine (20.5%) of 44 state health departments (California, Florida, Idaho, Kansas, Kentucky, Louisiana, Maryland, New Hampshire,  and Rhode Island) designated third-party organizations to receive and administer NPCR-awarded federal funds to operate their cancer registries. Of the 44 state programs receiving federal funds, 35 enhancement programs (79.5%) had an existing cancer registry at the time of NPCR funding, and the remaining 9 planning programs (20.5%) were newly established cancer registries. Thirty-three (75.0%) programs had 1999 incidence data that met the *United States Cancer Statistics* publication criteria for high-quality data. Six (13.6%) programs had one or more SEER Program registries operating within their catchment areas. Of the 44 state programs, 16 (36.4%) were in the South, 10 (22.7%) were in the Midwest, 10 (22.7%) were in the West, and 8 (18.2%) were in the Northeast. The average percentage of the population that lived in an urban area (based on the 1990 census) was 68.4%, with a range of 32.2% (Vermont) to 100.0% (District of Columbia). The average geographic area of the state programs was 7194 sq miles, with a range of 61.4 sq miles (District of Columbia) to 570,374 sq miles (Alaska).

The results (Table 3) from the full model (all variables included) revealed that the log average NPCR cost per case reported decreased as the log of the total number of annual cases reported increased (*P* ≤ .001). The log average NPCR cost per case reported increased as the geographic area of the state (expressed per 1000 sq miles) increased (*P* = .03). The results did not appreciably change when we did not include Alaska, the state with the largest geographic area in the analysis. The log average NPCR cost per case reported was lower (*P* = .07) for enhancement programs than for planning programs. The log average NPCR cost per case increased (*P* = .12) for registries as the cost of living in the state increased. The increase in the log average NPCR cost per case reported for states with data that met the criteria for reporting high-quality 1999 incidence data was not statistically significant (*P* = .65). Similarly, the log average annual NPCR cost per case reported was not associated (*P* = .66) with the percentage of the population living in an urban area. No association was found between the log average cost per case and 1) whether the state had a SEER metropolitan-area or special-population registry collecting and reporting cases within an NPCR catchment area (*P* = .78) or 2) whether the state health departments designated a third party to administer the cancer registry (*P* = .96).

We used results from the parsimonious regression model (Table 3), containing the log average annual number of cases reported, the type of funding (enhancement or planning), the regional CPI, and the geographic area of the state (per 1000 sq miles), to estimate the predicted log average annual cost per case for each program. The observed and predicted log average NPCR costs per case are shown in Figure 2. The cost per case reported decreased, whereas the log average number of cases reported increased. However, an examination of the predicted values shows that other factors need to be considered when predicting average NPCR costs.

## Discussion

During the first 5-year project period of the NPCR, notable progress was made toward establishing an infrastructure for nationwide cancer surveillance (27,28). In recent years, the NPCR's focus has broadened to include working with state and national partners to use collected data to target prevention, screening, and treatment services directed at high-risk populations (27). Improving the operational efficiencies of cancer registries will allow more state and federal resources to be used for cancer prevention and control — the original purpose of cancer registries. To guide program funding and management decisions and ensure the availability of funds for data analysis and interpretation, we must identify factors that influence the cost to report a case of cancer.

### Economies of scale

The major finding of this analysis is that the average NPCR cost to report a case of cancer decreases as output levels (i.e., incident cases) increase. Such an apparent economies-of-scale relationship was found in an economic analysis of the CDC's National Breast and Cervical Cancer Early Detection Program (NBCCEDP), in which the output measure was the number of women screened for breast and cervical cancer and the number of cancer cases detected through screening (29). As in the NBCCEDP analysis, the apparent existence of an economies-of-scale relationship in this analysis is most likely a result of fixed costs, such as administrative and infrastructure support. The average cost per case is lower when fixed costs are shared among more units of output. For example, each state program must provide administrative and technical support for the operation of the cancer registry — fixed costs that occur regularly, whether 1000 or 100,000 new cases of cancer are reported annually. Programs that report a higher number of incident cases experience a decrease in the average cost with their increase in output.

The average NPCR cost to report a case of cancer increases with increasing geographic coverage. Although cancer registries encourage electronic data reporting from outlying reporting facilities (e.g., hospital cancer registries, pathology laboratories, treatment facilities), routine surveillance and quality-assurance activities often involve visits from central cancer registry staff members to doctors' offices and hospitals to ensure that all incident cancer cases are reported accurately and in a timely manner. Increased geographic coverage may result in the hiring of additional staff members to coordinate regional activities. The compensation of the additional employees and their subsequent travel-related expenses increase the average cost to report a case.  

In addition, the average NPCR cost to report a case of cancer is higher in regions of the United States with higher costs of living (as measured by the regional CPI). General operating costs, including employee compensation, are higher in New England than in the South, where living costs are lower.

On average, incident cases of cancer cost less to report through an enhancement program than through a planning program. This finding is not surprising, given that enhancement programs receive maintenance and matching funds from their respective state health departments, whereas states are not required to provide financial support to planning programs.  In addition, enhancement programs were established before they began receiving NPCR funding, although we are unsure to what extent this affected the cost to report a case of cancer. As registries entered the second 5-year NPCR project period that began in 2000, nine state programs transitioned from receiving planning funding to receiving enhancement funding and began receiving financial or in-kind support from their state health departments as a condition of federal funding. Two states (South Dakota and Tennessee) and the three territories are still classified as planning programs in the current project period.

If state registry operations could be conducted more efficiently, more funds would be available for their state's cancer prevention and control activities. The existence of economies of scale in federally funded, state-managed surveillance programs suggests that states with fewer cases might benefit from integrating program services with neighboring states (e.g., by sharing resources and other fixed-cost expenses), depending on their geographic area and population size. In addition to reducing costs, sharing database management resources may promote uniform data collection and quality control practices while making it easier for states to share information about residents who receive diagnostic and treatment services outside their state of residence.

### Analysis limitations

This analysis has several limitations. Although we identified several important factors that seem to influence the average annual NPCR cost to report an incident case of cancer, we have underestimated the total cost for reporting a case of cancer. Comprehensive information on state support for the operation of cancer registries was not factored into our cost estimates. In addition to financial support, some states provide their registries with an indeterminate amount of direct or in-kind support from private and public health care facilities in the form of facility resources and staff time for surveillance-related activities. In addition, our NPCR costs do not include the total federal costs. Metropolitan-area and special-population cancer registries participating in the SEER Program received federal support through the NCI, and these funds were not included in our analyses. However, because the majority of NPCR-funded state programs presumably received the majority of their financial support from the CDC, the direction, if not the magnitude, of the relationships we observed are most likely correct.

Although the average NPCR cost to report a case of cancer seems to be higher in regions of the United States with higher costs of living, we may have underestimated the true impact of the CPI on registry operations. Given that the regions are large and geographically diverse, intraregional living costs may be as large as or larger than the interregional variability.

Because of the relatively small number of state programs (44 programs), this study may have lacked the statistical power to definitively identify all factors influencing costs. Although having a SEER Program registry within an NPCR-funded state and having high-quality incidence data were not significantly associated with the average NPCR cost to report a case of cancer through the state program, the direction of the coefficients suggests that these factors might help explain variations in cost per case reported. The coefficient for having a SEER Program registry within NPCR-funded cancer registries catchment areas was negative, suggesting a lower average NPCR cost when some of the cases are collected and reported through the SEER Program registry. Similarly, the coefficient for having data that met the criteria for inclusion in the *United States Cancer Statistics* report was positive, suggesting that additional costs are incurred when collecting and reporting high-quality incidence data.

A scatter plot comparison of the predicted and observed costs to report a case of cancer by each state program reveals that large variations exist among NPCR-funded state programs (Figure 2), and numerous registries are spending more or less than predicted to report a case of cancer. States that are spending more are not necessarily less efficient than those spending equal to or less than predicted. These states could be using NPCR funds for data-related cancer control and prevention activities as well as for core surveillance activities, all of which are encouraged by NPCR. Likewise, states with lower than predicted costs are not necessarily more efficient. Their registries could be receiving a larger proportion of their operating costs from their states. These observations merely suggest that additional analyses are needed to identify other factors to explain these discrepancies. In addition, in-depth analyses of individual state registries, which are currently underway at the CDC, are needed to estimate the total cost — including direct or in-kind support — to report a case of cancer.

## Figures and Tables

**Table 1:** Average Annual Funds Spent, Cancer Cases Reported, and Cost per Case Reported During the First 5-Year Project Period of the National Program of Cancer Registries (NPCR)

	**Mean/Median**	**SD**	**Range**
Average annual NPCR funds spent	$382,698/$312,698	$251,420	$120,464 (Delaware)-$1,080,370 (Texas)
Average annual number of cancer cases reported	27,353/18,999	28,843	1929 (Alaska)-132,525 (California)
Average annual NPCR cost per case reported	$29.20/$18.43	$38.71	$3.45 (Michigan)- $234.52 (Alaska)

**Table 2:** Registry and State Characteristics of Programs Participating in the National Program of Cancer Registries (NPCR)

**Characteristics**	**Frequency**	**Percentage**

**Categorical**		

**NPCR program administered by designee?**		

Yes	9	20.5
No	35	79.5

**NPCR program type**		

Enhancement (existing registry)	35	79.5
Planning (newly established registry)	9	20.5

**High-quality 1999 incidence data?**		

Yes	33	75.0
No	11	25.0

**Surveillance Epidemiology and End Results Program metropolitan area or special population within NPCR catchment area?**		

Yes	6	13.6
No	38	86.4

**2000 consumer price index**		

South (167.2)^a^	16	36.4
West (174.8)^a^	10	22.7
Midwest (168.3)^a^	10	22.7
Northeast (179.4)^a^	8	18.2

**Characteristics**	**Mean** **(SD)**	**Percentage Range**

**Continuous**		

Percentage of urban population (1990 census)	68.4 (15.4)	32.2 (Vermont)- 100.0 (District of Columbia)
Area (sq miles)	7194 (9073)	61.4 (District of Columbia)-570,374 (Alaska)

a Treated as a continuous variable in the analysis.

**Table 3:** Linear Regression Model of Log Average Cost per Case Reported for Selected Registry and State Characteristics of Programs Participating in the First 5-Year Project Period of the National Program of Cancer Registries (NPCR)

	**Coefficients** ** (95% CI; *P* value)^a^**	

**Explanatory Variables**	**Full Model**	**Parsimonious Model**
Log average annual cancer cases reported	–.571 (–0.715 to –0.427; <.001)	–.565 (–0.691 to –0.438; <.001)
Area (per 1000 sq miles)	.002 (0.000 to 0.004; .03)	.002 (0.001 to 0.004; .01)
NPCR program type (enhancement vs planning)	–.374 (–0.773 to 0.024; .07)	–.315 (–0.654 to 0.024; .08)
2000 consumer price index	.023 (–0.005 to 0.052; .12)	.025 (–0.002 to 0.051; .08)
High-quality 1999 incidence data	.079 (–0.262 to 0.421; .65)	NA
Percentage of urban population (1990 census)	<.002 (–0.008 to 0.013; .66)	NA
Surveillance Epidemiology and End Results Program metropolitan area or special population within NPCR catchment area	–.063 (–0.505 to 0.378; .78)	NA
NPCR program administered by designee	.010 (–0.337 to 0.356; .96)	NA

a Adjusted R^2^: full model, 0.73; parsimonious model, 0.71. NA indicates not applicable.
